# Meeting the ongoing challenges of outcome selection in surgical oncology trials

**DOI:** 10.1093/bjs/znac097

**Published:** 2022-04-12

**Authors:** Bilal Alkhaffaf, Jamie Kirkham

**Affiliations:** Department of Oesophago-Gastric and Bariatric Surgery, Salford Royal Hospital, Northern Care Alliance NHS Foundation Trust, Salford, UK; Division of Cancer Sciences, School of Medical Sciences, Faculty of Biology, Medicine and Health, University of Manchester, Manchester, UK; Centre for Biostatistics, University of Manchester, Manchester Academic Health Science Centre, Manchester, UK

## Introduction

Choosing appropriate outcomes to report in trials is an area of research faced with significant challenges, which have been highlighted extensively by clinicians and researchers. It is well established that the views and priorities of healthcare professionals are not necessarily shared by patients. If researchers decide to focus on outcomes that bear little relevance to key stakeholders such as patients, their studies risk contributing to the substantial levels of research waste endemic in the field of biomedical science^1^. Several studies have demonstrated considerable heterogeneity in the reporting of outcomes in surgical trials^2,3^. There is a lack of consistency with respect to which outcomes are reported and how they are measured, which makes evidence synthesis difficult and ultimately limits the degree to which trials can be translated into clinical practice. As an example, patient-reported outcomes (PROs), such as health-related quality of life (HR-QoL), have been promoted by some as an approach to better incorporating the lived experience of patients and caregivers into the assessment of medical interventions in clinical trials, yet their uptake has been extremely limited^4^.

One approach to addressing the variation in reporting is the development of core outcome sets (COSs)—an agreed standardized collection of outcomes that should be measured and reported, as a minimum, in all trials in a specific clinical area^5^. A COS directs trialists to which outcomes should be reported as a minimum, without restricting other outcomes that they may wish to select. Another key consideration is that COSs do not aim to define or select a single primary outcome to be studied within a given field; this decision should sit with the trial team and will change depending on the focus of the study. By incorporating the views of key stakeholders (including patients and care-givers with lived experience) in the selection of critically important outcomes to include in a COS, the relevance and quality of trials can be improved, regardless of who is undertaking them or where they are carried out.

The benefits of COSs have been widely recognized by grant awarding bodies, the US Food and Drug Administration, the European Medicines Agency, and Cochrane. These include the increased uptake of previously neglected PROs into trial design. Once the adoption of a COS within a particular field becomes established, the quality of data synthesis can be improved, which can ultimately drive evidence-based decision-making. This is a priority in areas such as surgical oncology where many patients have seen little improvement in survival over the past five decades^6^. Currently, there are five COSs available for use in surgical oncology (gastric, oesophageal, prostate, colorectal, and oropharyngeal cancers)^7–11^. There are no COSs for 16 of the commonest cancers worldwide that are treated surgically (such as breast, lung, renal)^12^, exposing a potential evidence gap and highlighting the work still required in these specialties.

### Emerging challenges in core outcome set development and adoption

Several challenges have been identified that must be met to fill this evidence gap. COSs can be time-consuming and resource-intensive, with many studies taking years to develop from project inception to publication. Although guidance and standards exist to support COS developers in delivering and reporting their projects^13–15^, further methodological refinement is required to streamline the process. This demand has been particularly highlighted by the COVID-19 pandemic, during which trials exploring innovative treatments have required the urgent development of a COS to help direct outcome selection. Financial considerations are also paramount as research funding continues to be rationalized. For example, at the time of writing, the UK’s National Institute for Health Research had awarded €4.7 million to fund the development of 14 COSs. In a review^16^ of Cochrane Review Group editors, the most frequently cited barriers to COS development were lack of time, resources, and funding. Clearly, approaches to make COSs cheaper and quicker to deliver are required.

Surgical trials have traditionally focused on the reporting of short-term outcomes, including complications. However, the way in which adverse events are considered and included in COSs have posed a unique challenge. Across all disciplines, less than one-third of COSs include an adverse event outcome. Furthermore, among the five COSs for surgical oncology trials, there is considerable variation in how harms are presented: whether these are pooled to include all serious complications, or whether selected complications (such as anastomotic leak in colorectal and oesophageal COSs) should be reported as a minimum.

Finally, unless COSs are deemed widely applicable within their respective clinical area, they will not be adopted by researchers and ultimately fail to achieve their intended benefit. Ironically, this can lend COSs to contribute to the research waste that they set out to address. It is not currently known how well COSs are being adopted as there have been only a handful of studies assessing this topic^17^. Although several initiatives exist to promote COS adoption^18^, this will be an area that needs focus in the future.

### Addressing challenges to benefit surgical cancer trials

Decision-making in the clinical setting is a process that carefully considers the balance of benefits and harms. This balance should be reflected in the development of COSs, and consequently both benefits and harms should be routinely included in them. Furthermore, most COSs focus on a particular health condition. Although this is clearly beneficial when designing trials for the disease in question, it does little to address the wider challenges highlighted above. From the examination of current COSs for surgical oncology trials (*Table 1*), there was significant overlap with respect to the outcomes included. For example, all five include outcomes related to survival, impacts on HR-QoL, and adverse events. The majority also include gastrointestinal or nutritional effects and an assessment of completeness of surgical resection. These similarities demonstrate sufficient overlap to justify the development of a meta-COS for surgical oncology trials. This first-of-its-kind approach will build on lessons learnt from previous COSs in this field to develop a minimum set of outcomes relevant to key stakeholders, regardless of cancer type. Given the real need to address outcome selection across several areas in the field of cancer surgery research, such a meta-COS would negate the need for multiple protracted, financially costly, and resource-intensive studies every time trialists seek to select relevant trial endpoints.

**Table 1:** Outcomes included in core outcome sets for effectiveness trials in cancer surgery

	Outcome domain	Gastric cancer	Oesophageal cancer	Colorectal cancer	Prostate cancer	Oropharyngeal cancer
**Surviving and controlling cancer**	Overall survival		Overall survival	Long-term survival	Death from any cause	
	Disease-free survival	Disease-free survival			Disease progression	
	Disease-specific survival	Disease-specific survival			Death from prostate cancer	Disease-specific survival
	Recurrence	Recurrence of cancer		Cancer recurrence	Distant disease recurrence/metastases Local disease recurrence	Distant metastatic control
						Local control
						Regional control
	Surgery-related death	Surgery-related death	In-hospital mortality	Perioperative survival	Perioperative deaths	Death related to treatment
	Completeness of tumour removal	Completeness of tumour removal	Inoperability	Resection margins	Positive surgical margin	
	Need for additional cancer surgery				Need for salvage therapy	
**Adverse events**	Adverse events	Serious adverse events	Conduit necrosis and anastomotic leak	Anastomotic leak	Thromboembolic disease Bothersome or symptomatic urethral or anastomotic stricture	Interventions for management of treatment-related morbidity
				Surgical-site infection		
			Need for another operation related to primary oesophageal cancer resection surgery	Stoma rates and complications		
				Conversion to open operation (where appropriate)		
			Respiratory complications			
**Impacts of surgery**	Impact on quality of life	Overall quality of life	Overall quality of life	Physical function	Overall quality of life	Health-related quality of life
				Sexual function	Sexual function	
	Impact on nutrition	Nutritional effects	Severe nutritional problems			
			Ability to eat and drink			
	Gastrointestinal impacts		Problems with acid indigestion or heartburn	Faecal incontinence	Bowel function	Dysphagia
				Faecal urgency	Faecal incontinence	
					Stress incontinence	
					Urinary function	

Once there is agreement on which outcomes should be included in a COS, approaches to identify appropriate outcome measurement instruments (OMIs) can commence. Although a COS in and of itself can provide guidance with respect to which outcome domains should be reported as a minimum, it is important that researchers are also given guidance with respect to the OMIs that can be used to report them. The process of selecting OMIs should be as methodologically robust as determining which outcomes to measure. Although guidance exists with respect to how this may be done^19^, only a minority of COS studies (and none in cancer surgery) has moved on to this stage^18^. Some of these OMIs may be clinician-reported, observer-reported or patient-reported (for example, PROs such as HR-QoL)^20^. The topic of how outcomes can or should be measured is expansive and outside the scope of this article.

A broad collaborative approach is essential to facilitate the uptake of a (meta-)COS so that its benefits are realized, and researchers from across the wider field of COS development can consider it as a method for addressing the challenges outlined here. It may be argued that COS implementation can only really commence once consensus has been reached on both which outcomes should be reported and how they should be measured; undoubtedly, early and close collaboration between COS developers and stakeholders is required. Examples include inviting grant-awarding bodies, regulators, and journals that can promote COS use to participate in development of the COS either through management of the study and/or by participating in the development process. CROWN (CoRe Outcomes in Women’s and Newborn health) is an international initiative, led by journal editors, to harmonize outcome reporting in women’s health research (http://www.crown-initiative.org/); similar strategies should be employed by journals with an interest in surgical oncology.

In summary, outcome selection remains a challenge in the field of surgical oncology. This affects the relevance of trials, which has a negative impact on the implementation of effective treatments in clinical practice. However, through collaboration and leadership by key stakeholder groups to develop initiatives such as the approach described here, considerable inroads can be made in improving research quality and addressing these challenges.

*Disclosure*. The authors declare no conflict of interest.
